# Supplementary material for: Prospective Association of the Portfolio Diet with All-Cause and Cause-Specific Mortality Risk in the Mr. OS and Ms. OS Study
Source: Nutrients. 2021 Dec 3;13(12):4360. doi: 10.3390/nu13124360 (PMC8705939; doi:10.3390/nu13124360)
Supplement: Supplementary file 1 [file nutrients-13-04360-s001.zip › nutrients-1470119-supplementary.pdf]

Supplementary Figure S1 Cumulative probability of mortality due to cardiovascular disease (CVD) or cancer

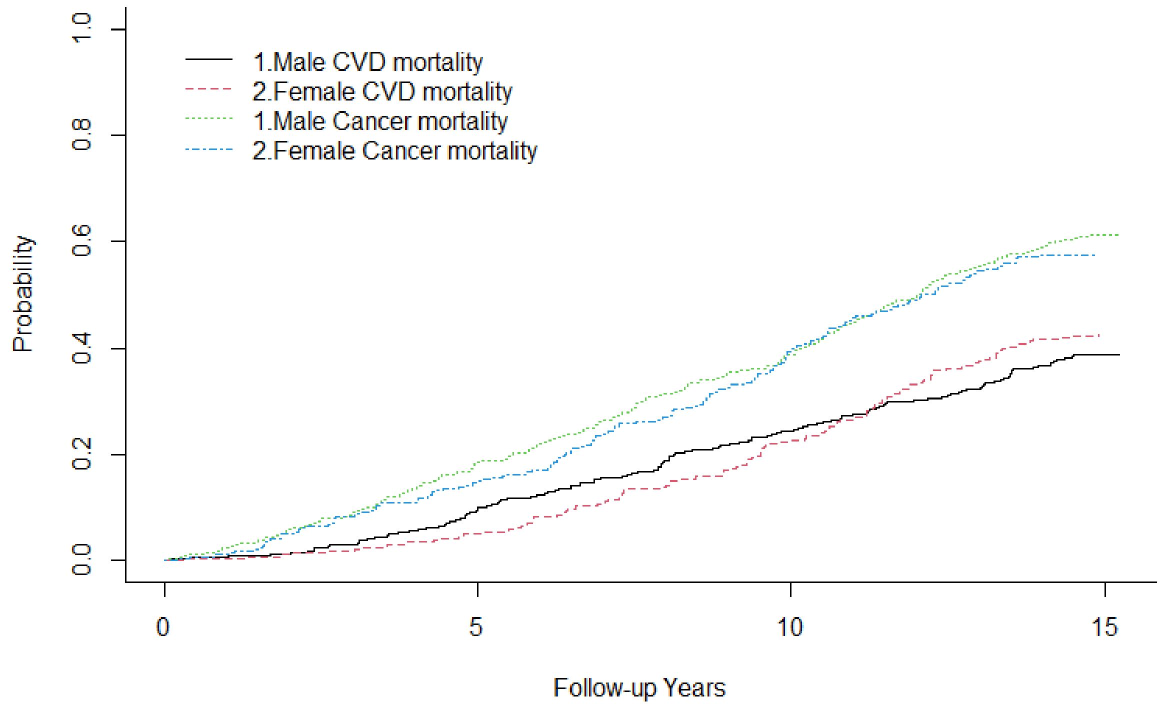

Supplementary Figure S2 Testing proportional Hazards assumption

	All participants	Males	Females
**All-cause mortality**	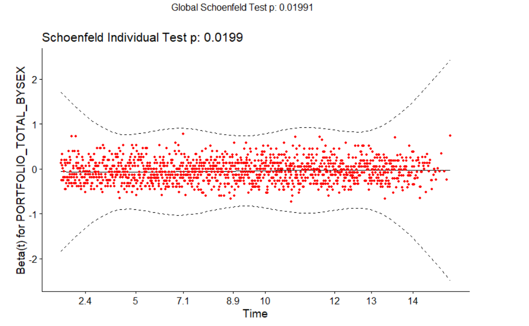	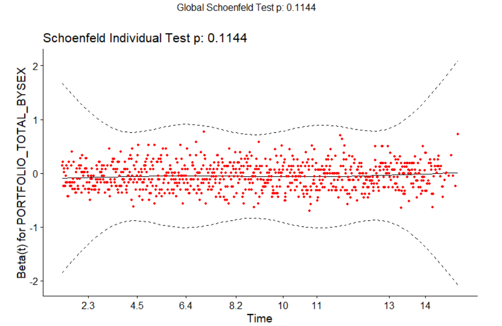	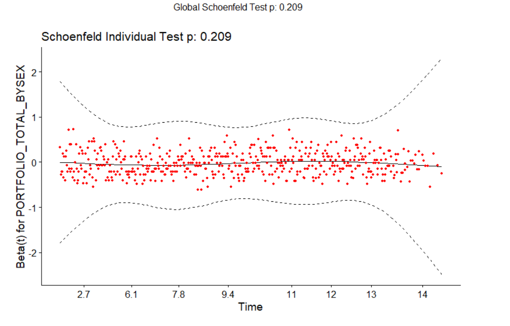
**CVD mortality**	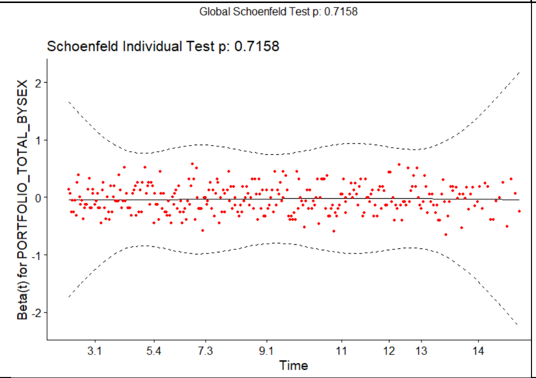	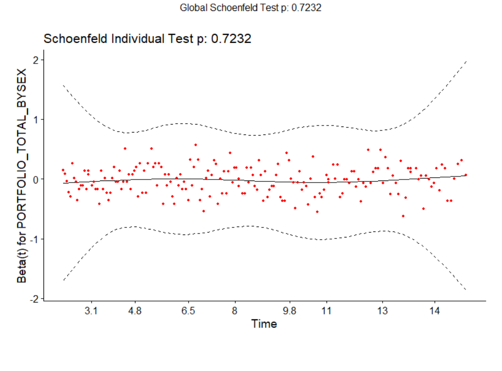	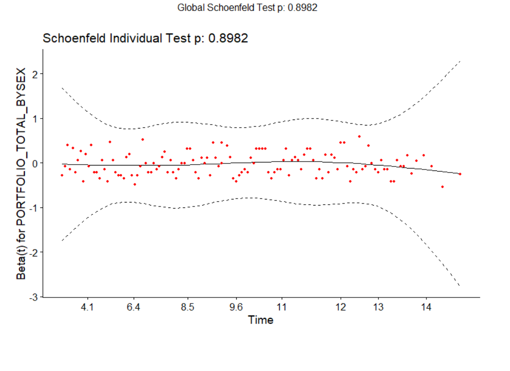
**Cancer mortality**	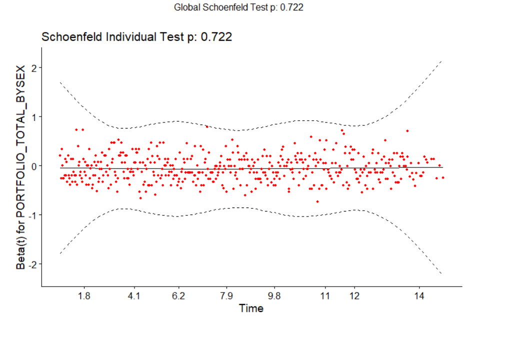	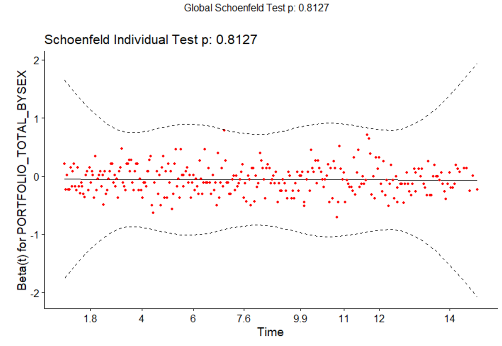	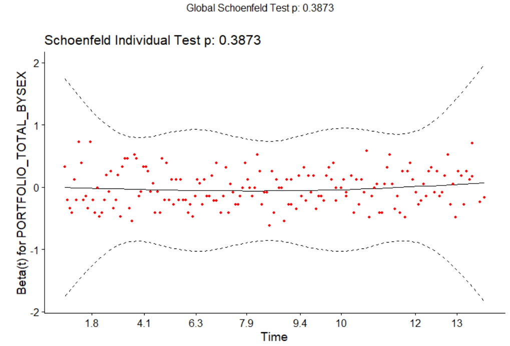

Supplementary Table S1 Comparing the Portfolio Diet score components in male and female participants

Component	Quintile 1, servings/day#	Quintile 2, servings/day#	Quintile 3, servings/day#	Quintile 4, servings/day#	Quintile 5, servings/day#
**Male participants**					
Plant protein sources	1.297	2.795	4.249	6.237	11.393
Viscous fiber sources	0.643	1.864	3.038	4.261	8.237
Nuts	0.000^	0.015	0.041	0.088	0.329
Plant sterols#	176 mg	260 mg	345 mg	431 mg	596 mg
MUFAs sources	0.000^	0.000^	0.000^	0.000^	0.390
Saturated fat/cholesterol sources	0.667	1.266	1.866	2.665	4.854
**Female participants**					
Plant protein sources	1.081	2.114	3.174	4.737	9.070
Viscous fiber sources	0.995	2.607	3.792	5.312	8.556
Nuts	0.000^	0.003	0.025	0.057	0.219
Plant sterols#	151 mg	231 mg	296 mg	366 mg	559 mg
MUFAs sources	0.000^	0.000^	0.000^	0.108	0.374
Saturated fat/cholesterol sources	0.331	0.697	1.023	1.459	2.832

Abbreviation: MUFA, monounsaturated fatty acids.

Mean intake of plant sterols is presented in mg/day instead of servings/day for other components.

^ No points were assigned to participants due to very low consumption.

Supplementary Table S2 Sensitivity analysis on the associations between the highest to lowest adherence to the Portfolio Diet and risk of mortality

	All-cause mortality<br>HR (95% CI)	CVD mortality<br>HR (95% CI)	Cancer mortality<br>HR (95% CI)
**Male participants**			
Excluding people with diabetes	0.60 (0.47, 0.77) *	0.69 (0.41, 1.17)	0.61 (0.40, 0.93) *
Excluding people with hypertension	0.46 (0.33, 0.63) *	0.70 (0.36, 1.35)	0.44 (0.26, 0.75) *
Excluding people with stroke	0.62 (0.49, 0.77) *	0.87 (0.55, 1.37)	0.58 (0.38, 0.87) *
Excluding people with heart attack	0.66 (0.52, 0.84) *	0.94 (0.57, 1.54)	0.60 (0.39, 0.91) *
Excluding people with angina	0.63 (0.50, 0.79) *	0.90 (0.57, 1.43)	0.59 (0.39, 0.89) *
Excluding people with congestive heart failure	0.62 (0.50, 0.78) *	0.87 (0.56, 1.35)	0.58 (0.39, 0.88) *
Excluding people with cancer	0.66 (0.53, 0.83) *	0.96 (0.61, 1.49)	0.64 (0.42, 0.97) *
Excluding systolic blood pressure from the model	0.63 (0.51, 0.79) *	0.89 (0.58, 1.38)	0.59 (0.39, 0.87) *
Excluding people with ever smoked	0.48 (0.33, 0.70) *	0.51 (0.25, 1.02)	0.47 (0.23, 0.95) *
Using the age at death as survival time	0.60 (0.48, 0.75) *	1.10 (0.71, 1.69)	0.59 (0.40, 0.87) *
**Female participants**			
Excluding people with diabetes	0.92 (0.69, 1.24)	0.93 (0.51, 1.69)	0.83 (0.51, 1.33)
Excluding people with hypertension	0.62 (0.42, 0.92) *	0.72 (0.33, 1.56)	0.52 (0.27, 0.98) *
Excluding people with stroke	0.86 (0.65, 1.13)	0.87 (0.50, 1.50)	0.85 (0.55, 1.32)
Excluding people with heart attack	0.83 (0.62, 1.12)	0.84 (0.46, 1.56)	0.79 (0.50, 1.25)
Excluding people with angina	0.88 (0.67, 1.17)	0.90 (0.51, 1.58)	0.91 (0.58, 1.43)
Excluding people with congestive heart failure	0.91 (0.69, 1.20)	0.90 (0.53, 1.55)	0.92 (0.59, 1.42)
Excluding people with cancer	0.91 (0.69, 1.20)	1.00 (0.59, 1.71)	0.92 (0.58, 1.44)
Excluding systolic blood pressure from the model	0.88 (0.67, 1.15)	0.91 (0.54, 1.55)	0.87 (0.57, 1.34)
Excluding people with ever smoked	0.92 (0.69, 1.22)	1.01 (0.57, 1.78)	0.86 (0.55, 1.37)
Using the age at death as survival time	0.85 (0.65, 1.11)	0.93 (0.55, 1.57)	0.84 (0.54, 1.31)

Abbreviations: CVD, cardiovascular disease; HR, hazard ratio. *$p<0.05$.

The Cox regression model was adjusted for sex, age, dietary energy, body mass index, physical activity, systolic blood pressure, medical history (diabetes, hypertension, stroke, heart attack, angina, congestive heart failure or cancer), smoking habit, alcohol drinking, education level.

Supplementary Table S3 Competing risk analysis on the association between Portfolio Diet Score and the Risk of CVD or Cancer Mortality

	**CVD mortality** SHR (95% CI)	**Cancer mortality** SHR (95% CI)
**Male**		
Q1 (N=620)	1.00	1.00
Q2 (N=405)	0.99 (0.72, 1.36)	0.84 (0.66, 1.07)
Q3 (N=576)	1.02 (0.76, 1.38)	0.88 (0.52, 0.93) *
Q4 (N=395)	1.02 (0.73, 1.44)	0.54 (0.43, 0.67) *
**Female**		
Q1 (N=548)	1.00	1.00
Q2 (N=539)	0.77 (0.48, 1.24)	0.64 (0.42, 0.97) *
Q3 (N=504)	0.80 (0.49, 1.31)	0.60 (0.39, 0.93) *
Q4 (N=404)	0.92 (0.54, 1.56)	0.84 (0.54, 1.31)

Abbreviations: CVD, cardiovascular disease; SHR, sub-distribution hazard ratio. *$p<0.05$.
The Fine-Gray model was adjusted for sex, age, dietary energy, body mass index, physical activity, systolic blood pressure, medical history (diabetes, hypertension, stroke, heart attack, angina, congestive heart failure or cancer), smoking habit, alcohol drinking, education level.
